# 1-De­oxy-l-mannitol (6-de­oxy-l-mannitol or l-rhamnitol)

**DOI:** 10.1107/S1600536808024586

**Published:** 2008-08-06

**Authors:** Sarah F. Jenkinson, K. Victoria Booth, Pushpakiran Gullapalli, Kenji Morimoto, Ken Izumori, George W. J. Fleet, David J. Watkin

**Affiliations:** a Department of Organic Chemistry, Chemistry Research Laboratory, University of Oxford, Mansfield Road, Oxford OX1 3TA, England; b Rare Sugar Research Centre, Kagawa University, 2393 Miki-cho, Kita-gun, Kagawa 761-0795, Japan; c Department of Chemical Crystallography, Chemistry Research Laboratory, University of Oxford, Mansfield Road, Oxford OX1 3TA, England

## Abstract

The crystalline form of 1-de­oxy-l-mannitol, C_6_H_14_O_5_, exists as an extensively hydrogen-bonded structure with each mol­ecule acting as a donor and acceptor for five hydrogen bonds. There are no unusual crystal-packing features; the absolute configuration was determined from the use of 6-de­oxy-l-mannose (l-rhamnose) as the starting material.

## Related literature

For related literature see: Jenkinson *et al.* (2008 ▶); Gullapalli *et al.* (2007 ▶); Izumori (2002 ▶, 2006 ▶); Granstrom *et al.* (2004 ▶); Beadle *et al.* (1992 ▶); Skytte (2002 ▶); Sui *et al.* (2005 ▶); Levin (2002 ▶); Howling & Callagan (2000 ▶); Bertelsen *et al.* (1999 ▶); Takata *et al.* (2005 ▶); Menavuvu *et al.* (2006 ▶); Hossain *et al*. (2006 ▶); Donner *et al.* (1999 ▶).
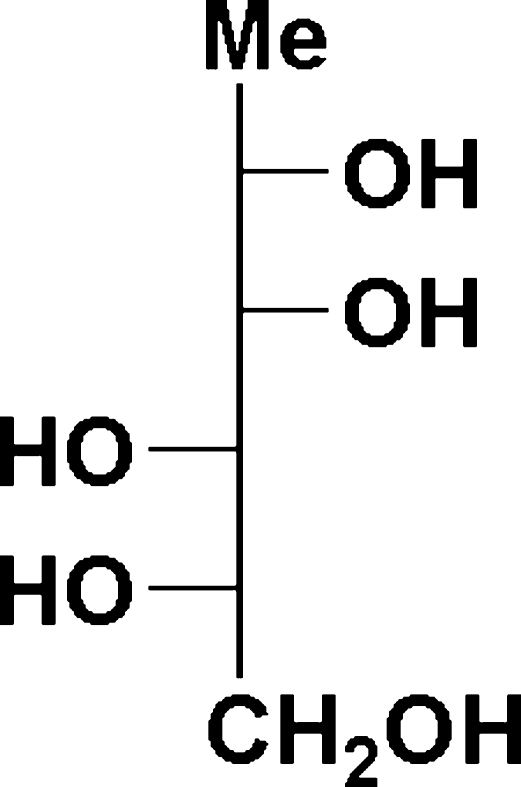


## Experimental

### 

#### Crystal data

C_6_H_14_O_5_
*M* *_r_* = 166.17
Orthorhombic, 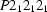

*a* = 7.3650 (3) Å
*b* = 7.6272 (3) Å
*c* = 13.7676 (5) Å
*V* = 773.39 (5) Å^3^
*Z* = 4
Mo *K*α radiation
μ = 0.12 mm^−1^
*T* = 150 K
0.40 × 0.40 × 0.10 mm

#### Data collection

Nonius KappaCCD diffractometer
Absorption correction: multi-scan (*DENZO*/*SCALEPACK*; Otwinowski & Minor, 1997 ▶) *T*_min_ = 0.89, *T*_max_ = 0.99
5170 measured reflections
1033 independent reflections
974 reflections with *I* > 2σ(*I*)
*R*_int_ = 0.024

#### Refinement

*R*[*F* ^2^ > 2σ(*F* ^2^)] = 0.027
*wR*(*F* ^2^) = 0.072
*S* = 0.97
1033 reflections
100 parameters
H-atom parameters constrained
Δρ_max_ = 0.24 e Å^−3^
Δρ_min_ = −0.19 e Å^−3^

### 

Data collection: *COLLECT* (Nonius, 2001 ▶); cell refinement: *DENZO*/*SCALEPACK* (Otwinowski & Minor, 1997 ▶); data reduction: *DENZO*/*SCALEPACK*; program(s) used to solve structure: *SIR92* (Altomare *et al.*, 1994 ▶); program(s) used to refine structure: *CRYSTALS* (Betteridge *et al.*, 2003 ▶); molecular graphics: *CAMERON* (Watkin *et al.*, 1996 ▶); software used to prepare material for publication: *CRYSTALS*.

## Supplementary Material

Crystal structure: contains datablocks global, I. DOI: 10.1107/S1600536808024586/lh2670sup1.cif

Structure factors: contains datablocks I. DOI: 10.1107/S1600536808024586/lh2670Isup2.hkl

Additional supplementary materials:  crystallographic information; 3D view; checkCIF report

## Figures and Tables

**Table 1:** Hydrogen-bond geometry (Å, °)

*D*—H⋯*A*	*D*—H	H⋯*A*	*D*⋯*A*	*D*—H⋯*A*
O10—H1⋯O1^i^	0.85	1.98	2.782 (2)	158
O4—H2⋯O6^ii^	0.87	1.92	2.779 (2)	168
O8—H3⋯O4^ii^	0.84	1.97	2.742 (2)	152
O6—H4⋯O10^iii^	0.87	1.92	2.772 (2)	165
O1—H5⋯O8^i^	0.87	1.84	2.704 (2)	173

Symmetry codes: (i) 
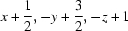
; (ii) 
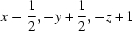
; (iii) 
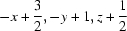
.

**Fig. 1.**
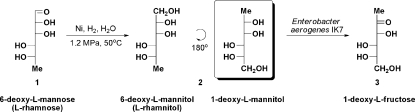
Synthetic scheme.

**Fig. 2.**
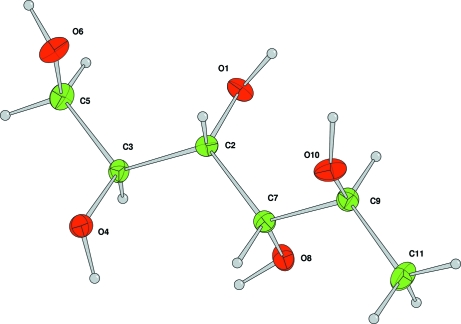
The title compound with displacement ellipsoids drawn at the 50% probability level. H atoms are shown as spheres of arbitary radius.

**Fig. 3.**
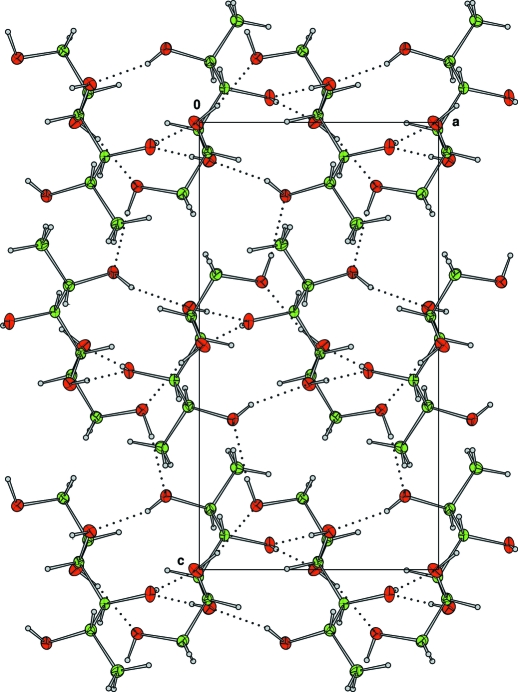
Packing diagram for the title compound projected along the b axis. Hydrogen bonds are shown as dotted lines.

## supplementary crystallographic information

### Comment

The properties of 1-deoxy ketohexose sugars have been little studied. The crystal structure of 6-deoxy-L-galactitol has recently been published (Jenkinson *et al.*, 2008) and herein we report the crystal structure of a similar deoxy polyol, 1-deoxy-L-mannitol an intermediate in the synthesis of 1-deoxy-L-fructose, 3 (Fig. 1) (Gullapalli *et al.*, 2007).

The demand for the large scale production of rare sugars by biotechnological (Izumori, 2006; Izumori, 2002; Granstrom *et al.*, 2004) and chemical (Beadle *et al.*, 1992) methods is driven by the demand for alternative foodstuffs (Skytte, 2002) and D-tagatose itself is used as a low calorie sweetener (Levin, 2002; Howling & Callagan, 2000; Bertelsen *et al.* 1999). Rare monosaccharides themselves, however, have been found to demonstrate interesting pharmaceutical properties, for example, D-psicose (Takata *et al.*, 2005; Menavuvu *et al.*, 2006) and D-allose (Sui *et al.*, 2005; Hossain *et al.*, 2006) have significant chemotherapeutic properties and D-tagatose has been found to be an anti-hyperglycemic agent (Donner *et al.*, 1999) and therefore potentially useful in the treatment of diabetes.

1-Deoxy-L-mannitol 2 (Fig. 2) was prepared from the reduction by catalytic hydrogenation of 6-deoxy-L-mannose 1 (L-rhamnose). The X-ray structure shows that the crystal exists as an extensively hydrogen bonded lattice with each molecule acting as a donor and an acceptor for 5 hydrogen bonds (Fig.3).

### Experimental

1-Deoxy-L-mannitol was recrystallized from methanol: m.p. 390K, [α]_D_^20^ +1.4 (*c*, 1.4 in H_2_O).

### Refinement

In the absence of significant anomalous scattering, Friedel pairs were merged and the absolute configuration was determined from the starting material.

The H atoms were all located in a difference map, but those attached to carbon atoms were repositioned geometrically. The H atoms were initially refined with soft restraints on the bond lengths and angles to regularize their geometry (C—H in the range 0.93–0.98, O—H = 0.82 Å) and *U*_iso_(H) (in the range 1.2–1.5 times *U*_eq_ of the parent atom), after which the positions were refined with riding constraints.

### Crystal data

**Table:**

C_6_H_14_O_5_	*F*_000_ = 360
*M**_r_* = 166.17	*D*_x_ = 1.427 Mg m^−^^3^
Orthorhombic, *P*2_1_2_1_2_1_	Mo *K*α radiation λ = 0.71073 Å
Hall symbol: P 2ac 2ab	Cell parameters from 1002 reflections
*a* = 7.3650 (3) Å	θ = 5–27º
*b* = 7.6272 (3) Å	µ = 0.12 mm^−^^1^
*c* = 13.7676 (5) Å	*T* = 150 K
*V* = 773.39 (5) Å^3^	Plate, colourless
*Z* = 4	0.40 × 0.40 × 0.10 mm

### Data collection

**Table:**

Nonius KappaCCD diffractometer	974 reflections with *I* > 2σ(*I*)
Monochromator: graphite	*R*_int_ = 0.024
*T* = 150 K	θ_max_ = 27.5º
ω scans	θ_min_ = 5.2º
Absorption correction: multi-scan(DENZO/SCALEPACK; Otwinowski & Minor, 1997)	*h* = −9→9
*T*_min_ = 0.89, *T*_max_ = 0.99	*k* = −9→9
5170 measured reflections	*l* = −17→17
1033 independent reflections	

### Refinement

**Table:**

Refinement on *F*^2^	Hydrogen site location: inferred from neighbouring sites
Least-squares matrix: full	H-atom parameters constrained
*R*[*F*^2^ > 2σ(*F*^2^)] = 0.027	*w* = 1/[σ^2^(*F*^2^) + (0.04*P*)^2^ + 0.19*P*], where *P* = [max(*F*_o_^2^,0) + 2*F*_c_^2^]/3
*wR*(*F*^2^) = 0.072	(Δ/σ)_max_ = 0.0003
*S* = 0.97	Δρ_max_ = 0.24 e Å^−^^3^
1033 reflections	Δρ_min_ = −0.19 e Å^−^^3^
100 parameters	Extinction correction: None
Primary atom site location: structure-invariant direct methods	

### Fractional atomic coordinates and isotropic or equivalent isotropic displacement parameters (Å^2^)

**Table:**

	*x*	*y*	*z*	*U*_iso_*/*U*_eq_	
O1	0.45760 (15)	0.66827 (14)	0.58528 (7)	0.0158	
C2	0.5038 (2)	0.53406 (18)	0.51734 (10)	0.0121	
C3	0.4654 (2)	0.35710 (19)	0.56608 (11)	0.0129	
O4	0.51432 (16)	0.21669 (13)	0.50177 (8)	0.0180	
C5	0.5694 (2)	0.3334 (2)	0.65961 (11)	0.0160	
O6	0.76010 (15)	0.34756 (16)	0.64310 (8)	0.0190	
C7	0.3954 (2)	0.55797 (19)	0.42326 (11)	0.0125	
O8	0.20579 (15)	0.57629 (14)	0.44513 (8)	0.0163	
C9	0.4543 (2)	0.7196 (2)	0.36498 (10)	0.0140	
O10	0.63971 (16)	0.69611 (16)	0.33563 (8)	0.0188	
C11	0.3428 (3)	0.7388 (2)	0.27300 (11)	0.0195	
H21	0.6338	0.5422	0.5017	0.0146*	
H31	0.3366	0.3507	0.5836	0.0149*	
H51	0.5258	0.4239	0.7048	0.0180*	
H52	0.5424	0.2171	0.6890	0.0191*	
H71	0.4147	0.4569	0.3816	0.0137*	
H91	0.4402	0.8236	0.4059	0.0171*	
H111	0.3791	0.8390	0.2343	0.0290*	
H112	0.2112	0.7500	0.2863	0.0299*	
H113	0.3580	0.6334	0.2330	0.0284*	
H1	0.7159	0.7532	0.3689	0.0319*	
H2	0.4249	0.1898	0.4627	0.0307*	
H3	0.1795	0.4708	0.4542	0.0290*	
H4	0.8002	0.3523	0.7025	0.0312*	
H5	0.5310	0.7560	0.5771	0.0285*	

### Atomic displacement parameters (Å^2^)

**Table:**

	*U*^11^	*U*^22^	*U*^33^	*U*^12^	*U*^13^	*U*^23^
O1	0.0168 (6)	0.0130 (5)	0.0175 (5)	−0.0022 (4)	0.0027 (5)	−0.0036 (4)
C2	0.0103 (7)	0.0137 (6)	0.0122 (6)	0.0004 (6)	0.0007 (6)	−0.0009 (5)
C3	0.0118 (7)	0.0125 (6)	0.0145 (7)	0.0011 (6)	0.0000 (6)	0.0014 (5)
O4	0.0207 (6)	0.0137 (5)	0.0198 (5)	0.0040 (5)	−0.0064 (5)	−0.0030 (4)
C5	0.0146 (8)	0.0191 (7)	0.0144 (7)	0.0011 (6)	0.0020 (6)	0.0022 (6)
O6	0.0144 (6)	0.0283 (6)	0.0142 (5)	0.0026 (5)	−0.0015 (4)	−0.0002 (4)
C7	0.0103 (7)	0.0127 (7)	0.0146 (7)	0.0004 (5)	0.0003 (6)	−0.0003 (6)
O8	0.0102 (5)	0.0128 (5)	0.0259 (6)	0.0000 (4)	−0.0005 (4)	0.0033 (4)
C9	0.0130 (7)	0.0143 (6)	0.0148 (7)	−0.0010 (6)	0.0003 (6)	0.0017 (6)
O10	0.0120 (6)	0.0284 (6)	0.0160 (5)	−0.0049 (5)	0.0009 (4)	−0.0020 (5)
C11	0.0173 (8)	0.0250 (8)	0.0163 (7)	0.0002 (7)	−0.0017 (6)	0.0065 (6)

### Geometric parameters (Å, °)

**Table:**

O1—C2	1.4277 (17)	O6—H4	0.870
O1—H5	0.868	C7—O8	1.4354 (18)
C2—C3	1.5335 (19)	C7—C9	1.533 (2)
C2—C7	1.5323 (19)	C7—H71	0.971
C2—H21	0.983	O8—H3	0.837
C3—O4	1.4354 (18)	C9—O10	1.4352 (19)
C3—C5	1.509 (2)	C9—C11	1.516 (2)
C3—H31	0.980	C9—H91	0.979
O4—H2	0.875	O10—H1	0.845
C5—O6	1.4269 (18)	C11—H111	0.969
C5—H51	0.983	C11—H112	0.991
C5—H52	0.995	C11—H113	0.981
			
C2—O1—H5	108.6	C2—C7—O8	109.94 (12)
O1—C2—C3	107.49 (11)	C2—C7—C9	112.99 (12)
O1—C2—C7	110.15 (12)	O8—C7—C9	107.87 (12)
C3—C2—C7	112.26 (12)	C2—C7—H71	109.2
O1—C2—H21	109.3	O8—C7—H71	110.1
C3—C2—H21	109.3	C9—C7—H71	106.7
C7—C2—H21	108.4	C7—O8—H3	99.4
C2—C3—O4	109.91 (11)	C7—C9—O10	108.44 (13)
C2—C3—C5	112.67 (12)	C7—C9—C11	111.20 (12)
O4—C3—C5	108.04 (12)	O10—C9—C11	106.98 (12)
C2—C3—H31	109.3	C7—C9—H91	108.7
O4—C3—H31	111.0	O10—C9—H91	111.4
C5—C3—H31	105.9	C11—C9—H91	110.2
C3—O4—H2	111.5	C9—O10—H1	114.5
C3—C5—O6	110.76 (12)	C9—C11—H111	112.7
C3—C5—H51	106.9	C9—C11—H112	112.6
O6—C5—H51	111.7	H111—C11—H112	107.7
C3—C5—H52	110.6	C9—C11—H113	109.2
O6—C5—H52	109.2	H111—C11—H113	107.8
H51—C5—H52	107.6	H112—C11—H113	106.6
C5—O6—H4	100.8		

### Hydrogen-bond geometry (Å, °)

**Table:**

*D*—H···*A*	*D*—H	H···*A*	*D*···*A*	*D*—H···*A*
O10—H1···O1^i^	0.85	1.98	2.782 (2)	158
O4—H2···O6^ii^	0.87	1.92	2.779 (2)	168
O8—H3···O4^ii^	0.84	1.97	2.742 (2)	152
O6—H4···O10^iii^	0.87	1.92	2.772 (2)	165
O1—H5···O8^i^	0.87	1.84	2.704 (2)	173

Symmetry codes: (i) *x*+1/2, −*y*+3/2, −*z*+1; (ii) *x*−1/2, −*y*+1/2, −*z*+1; (iii) −*x*+3/2, −*y*+1, *z*+1/2.
